# Overview of Updated Control Tactics for Western Flower Thrips

**DOI:** 10.3390/insects14070649

**Published:** 2023-07-20

**Authors:** Daniel Rodríguez, Ericsson Coy-Barrera

**Affiliations:** 1 Biological Control Laboratory, Facultad de Ciencias Básicas y Aplicadas, Universidad Militar Nueva Granada, Cajicá 250247, Colombia; 2 Bioorganic Chemistry Laboratory, Facultad de Ciencias Básicas y Aplicadas, Universidad Militar Nueva Granada, Cajicá 250247, Colombia

**Keywords:** *Frankliniella occidentalis* Pergande, management, biological control, ethological control

## Abstract

**Simple Summary:**

The western flower thrips (WFT), *Frankliniella occidentalis* Pergande, is a significant agricultural pest that challenges crop production. WFT are tiny insects that feed on various plants, causing damage that affects crop yield and quality. Effective control strategies are necessary to mitigate their impact. This review briefly overviews WFT and highlights key control strategies. Monitoring thrips populations through methods like sticky traps and visual inspections is crucial for timely intervention and assessing infestation levels, while control strategies for WFT include crop rotation and diversification to disrupt their life cycle, as well as the removal of alternate host plants and weeds that serve as reservoirs. In addition, selecting the appropriate insecticides based on thrips’ susceptibility and minimal impact on beneficial insects, and integrated pest management approaches combining cultural practices and biological control, are also recommended. Implementing these control strategies helps manage WFT effectively, protect crop production, and maintain a sustainable agricultural system.

**Abstract:**

*Frankliniella occidentalis* Pergande (Thysanoptera: Thripidae), broadly known as Western flower thrips (WFT), are currently one of the most critical pests worldwide in field and greenhouse crops, and their management is full of yet unsolved challenges derived from their high reproductive potential, cryptic habit, and ability to disperse. The control of this pest relies widely on chemical control, despite the propensity of the species to develop resistance. However, significant advances have been produced through biological and ethological control. Although there has recently been a remarkable amount of new information regarding the management of this pest worldwide, there is no critical analysis of recent developments and advances in the attractive control tactics for WFT, constituting the present compilation’s aim. Hence, this narrative review provides an overview of effective control strategies for managing thrips populations. By understanding the pest’s biology, implementing monitoring techniques, accurately identifying the species, and employing appropriate control measures, farmers and researchers can mitigate the WFT impact on agricultural production and promote sustainable pest management practices.

## 1. Introduction

Western flower thrips (WFT), *Frankliniella occidentalis* Pergande (Thysanoptera: Thripidae), are small, winged insects that belong to the Thripidae family [1]. These pests are native to western North America but have spread to various parts of the world due to human activity, including the global trade of ornamental plants, and since WFT are highly adaptable they have become a significant agricultural pest, causing damage to a wide range of crops [2]. The WFT adult measures only about 1.2 to 1.5 mm in length, making them barely visible to the naked eye. Their slender bodies with fringed wings allow them to fly short distances and they are typically yellowish-brown in color, but may appear darker or lighter depending on environmental factors and life stage [3]. In addition, one of the notable characteristics of WFT is their piercing-sucking mouthparts [4]. Feeding by WFT can result in several plant problems, threatening crop health [5].

Controlling WFT can be challenging due to their small size, population density, and ability to develop resistance to pesticides. Consequently, integrated pest management (IPM) strategies are often recommended for their control [5]. These strategies involve a combination of cultural, biological, ethological, and/or chemical control methods [6]. Some common approaches include using beneficial arthropods such as predatory mites and minute pirate bugs, which feed on thrips, as well as the careful application of insecticides targeted at the vulnerable life stages of thrips [6]. Additionally, preventive measures like regular monitoring, crop rotation, and proper hygiene in greenhouses or agricultural fields can help reduce the likelihood of thrips infestations [2]. Thrips traps, sticky cards, or yellow sticky traps can be used to monitor and catch adult thrips, providing valuable information about population levels [7]. In this context, implementing effective IPM strategies helps to mitigate the damage caused by WFT and protect valuable crops. Thus, this narrative review compiles updated information on different control tactics for WFT and underscores the importance of their implementation to management and crop protection.

## 2. Biology and Behavior of Western Flower Thrips (WFT)

The WFT individuals possess distinct physical characteristics that contribute to their identification. Understanding these features is essential for accurately identifying and distinguishing them from other thrips species. WFT are tiny insects, measuring approximately 1.2 to 1.5 mm in length as adults [1]. Their diminutive size makes them barely visible to the naked eye. The WFT’s body is elongated and slender, with a distinctive cigar-like shape. It appears narrow and cylindrical, allowing the thrips to maneuver through plant tissues easily. Their coloration can vary, but they typically exhibit a yellowish-brown or pale-brown hue, but the intensity of the color may vary depending on factors such as age, environmental conditions, and life stage [8]. Adult WFT have two pairs of fringed wings, usually longer than their bodies. These wings are narrow and extend beyond the length of the abdomen, and the fringed edges of the wings help distinguish them from other thrips species [9]. In addition, the WFT antennae are relatively long and segmented. They are noticeably thicker at the base and taper to a slender point; therefore, the length and shape of the antennae can aid in differentiating WFT from similar-looking insects [10]. On the other hand, WFT possess relatively short legs compared to the length of their bodies, with each leg terminating in arolia. These specialized structures serve as cushioned pads that enable thrips to adhere to plant surfaces effectively [11]. WFT have piercing-sucking mouthparts adapted for piercing plant tissues and extracting plant sap. The mouthparts enable them to feed on the sap of various crops and ornamental plants [4]. These physical characteristics can serve as general identifiers; however, accurate WFT identification may require a hand lens or microscope due to their small size. Furthermore, distinguishing features may vary slightly among individual thrips specimens and populations, often called pest complex, i.e., biotypes and subspecies of *F. occidentalis* [12].

### 2.1. Life Cycle and Reproductive Behavior of WFT

The life cycle and reproductive behavior of WFT play a crucial role in their population dynamics and ability to rapidly infest crops. WFT have a rapid life cycle, with females capable of laying up to 60 eggs during their lifespan of about 30 days [13]. The eggs are laid within plant tissues, and upon hatching, the larvae emerge and undergo two instars. Subsequently, they progress through the propupal and pupal stages before reaching adulthood. This quick reproductive cycle allows the thrips population to grow exponentially under favorable conditions [14].

The WFT life cycle begins with the oviposition. Female thrips insert their eggs into plant tissues, such as leaves, stems, or flowers [15]. The eggs are usually laid individually or in small groups. They are elongated, translucent, and extremely small, making them difficult to observe without magnification. After an incubation period of a few days, the eggs hatch into larvae. The larvae go through two stages, commonly called first and second instar [16]. During these stages, the larvae resemble smaller versions of the adults but lack wings. The WFT larvae typically display a pale yellow or white coloration, complemented by red eyes. Distinguishing between first and second-instar larvae can be achieved by examining the position and number of abdominal setae. Vierbergen et al. [17] identified specific characteristics of the WFT second-instar larvae, such as the presence of well-developed posteromarginal teeth measuring 5–7 μm in length in the abdominal tergite IX. These teeth are at least twice as long as the width of the D1 setae. Additionally, plaques found in the abdominal tergites II–V either lack microtrichiae or, if present, do not exceed 1 μm in length [17]. Following the larval stages, individuals enter the propupal stage and then progress to the pupal stage. During these stages, individuals cease feeding, and although they are not actively mobile, they may exhibit some limited movement if disturbed. Both pupal stages exhibit a cream or white coloration. Prepupae display short wing buds and forward-protruding antennae from the head. In pupae, the wing buds are more developed, often extending over halfway along the abdomen, while the antennae curve backward over the head [18]. During pupation, the individuals undergo metamorphosis, transforming into adult thrips. The pupal stage is generally short, lasting a few days [19]. After emerging from the pupal stage, WFT reach adulthood. Adult thrips are sexually mature and capable of reproduction. They possess two pairs of fringed wings, elongated bodies, and distinct coloration, typically yellowish-brown or pale brown [9].

WFT’s reproductive strategy enables them to multiply rapidly under favorable conditions. After reaching adulthood, WFT males and females engage in mating behavior. Male aggregations are a key aspect of the WFT mating behavior, usually observed on flowers. Within these aggregations, males emit an aggregation pheromone that attracts both males and females [20]. Females are observed to fly towards the aggregations, mate with a male, and then leave [21]. While males often engage in fighting behavior within the aggregations, the clear advantage of this behavior remains unclear, as females that arrive in the aggregations seem to mate with the first male they encounter. Indeed, experienced males are strongly inclined to mate with virgin females while actively avoiding mating with previously mated females [22]. Female thrips are highly fecund and can lay a significant number of eggs during their lifespan (ca. 45 days). For instance, on average, a single female thrips can lay up to 60–80 eggs [23]. The eggs are deposited into plant tissues using the female’s ovipositor. Various factors, including temperature, host plant quality, availability of suitable oviposition sites, and population density, influence the WFT reproductive rate [24,25]. Under optimal conditions, the population growth rate can be exponential, increasing thrips populations rapidly [1,2]. WFT are known for their ability to have a haplodiploid reproductive system, allowing for both sexual reproduction and arrhenotokous parthenogenesis. In sexual reproduction, fertilized eggs develop into females, while unfertilized eggs give rise to males [25]. This reproductive strategy allows females to produce offspring, potentially increasing their population growth rate. However, WFT females typically engage in monandry, mating with a single male, and they can re-mate five days after the initial mating event. Contrarily, males exhibit polygyny, having the capability to mate with multiple females [26]. This pattern of mating behavior results in a general bias towards females in WFT populations [27]. Understanding these aspects of their biology is essential for developing effective control strategies and implementing appropriate management practices.

### 2.2. Feeding Habits and Damage Caused by Thrips to Crops

WFT are notorious for their feeding habits and the damage they can cause to a wide range of crops [28]. Their piercing-sucking mouthparts can penetrate plant tissues and extract sap by a distinctive feeding strategy called “punch and suck” [29]. This feeding strategy involves the piercing of individual epidermal and mesophyll cells using their stylets, followed by the extraction of cell contents through sucking, and exhibiting sustained ingestion from unidentified plant tissues, potentially xylem sap [29,30], or the contents of previously ruptured cells [31]. Moreover, WFT engage in cell rupture feeding, as prolonged feeding by thrips leads to significant cell damage, including the destruction of cell walls, and they still feed on the released cell contents. This feeding behavior causes stippling/silvering, scarring, and discoloration of plant tissues. This damage appears as brown or bronze patches, streaks, or blotches on leaves, stems, and fruits [32]. WFT feeding on developing flowers can also lead to distorted or deformed blooms. Infested flowers may fail to open correctly or display abnormal shapes, and feeding on fruits can cause deformities, such as scars, pitting, or malformations, rendering them unmarketable or reducing their quality [33]. Severe thrips infestations can stunt plant growth and development, and consequently, the continual sap extraction weakens the plants, resulting in reduced vigor, decreased yields, and overall compromised plant health and fitness. In addition to causing direct feeding damage, WFT are significant vectors of plant viruses, e.g., the primary vector of tomato spotted wilt virus (TSWV) [34]. They can acquire the tospovirus from viruliferous plants within a well-defined interval, specifically during the first and early second larval instar stages. This period typically begins approximately 160 h after oviposition. During this phase, the insect undergoes ontogenetic changes characterized by the fusion of salivary gland cells with the mid-gut and visceral muscle cells. In late second instar larvae, the intimate contact between the salivary glands and visceral muscles is lost, reducing the movement of the virus within the salivary glands. In the metamorphosis process, the salivary glands separate from the mid-gut, effectively suppressing the virus’s movement into the salivary gland. Nevertheless, the individuals have already acquired the infection, and the virus continues to accumulate in the Malpighian tubules [35]. Upon reaching the adult stage, infected *F. occidentalis* can transmit the tospovirus to healthy plants during feeding events since, although the movement of the virus within the salivary glands is restricted in adults, the transmission can still occur due to the virus’s presence and activity in other anatomical structures [35]. This intricate pattern of virus acquisition, accumulation, and transmission highlights the role of WFT as vectors in the epidemiology of tospovirus diseases. Understanding these dynamics is crucial for devising effective management strategies to mitigate the transmission of tospoviruses by WFT and reduce the associated crop losses [36]. And finally, WFT have a broad host range and can affect numerous crops, including vegetables (e.g., tomatoes, peppers, cucumbers), fruits (e.g., strawberries, grapes), and various ornamental plants. Their ability to infest various crops makes them a significant concern for growers across different agricultural sectors [28].

## 3. WFT Monitoring

Monitoring WFT populations in the field is of utmost importance for effective pest management and timely intervention [1]. It allows for the early detection of WFT infestations since WFT populations can multiply rapidly, and early detection enables growers to identify and assess the severity of infestations before they escalate. Early intervention can help prevent population explosions and limit the damage caused by thrips. Moreover, monitoring provides crucial information for making informed decisions regarding pest control strategies [37]. By regularly monitoring thrips populations, growers can determine the population dynamics, assess the risk level, and decide on appropriate timing and intensity of control measures. WFT populations can exhibit seasonal fluctuations, with population peaks occurring during specific crop growth stages. Monitoring helps identify critical periods when crops are most susceptible to thrips damage [38]. This information enables growers to time their control measures effectively, ensuring maximum efficacy and minimizing potential yield losses. This information helps optimize resource allocation and minimize unnecessary pesticide use since regular monitoring allows for the early detection of insecticide resistance in thrips populations. WFT have demonstrated the ability to develop resistance to various insecticides. By monitoring populations for signs of resistance, growers can adjust their control strategies, rotate or alternate insecticides, and employ alternative methods to manage thrips effectively [39].

Monitoring helps establish population thresholds, which are predetermined levels of pest abundance that trigger the implementation of control measures. By regularly monitoring thrips populations and comparing them to established thresholds, growers can determine when intervention is necessary. This approach ensures that control measures are only deployed when populations exceed economically or ecologically significant levels. Indeed, monitoring is an integral component of IPM programs, which emphasize the use of multiple strategies for effective pest management [15]. By monitoring thrips populations, growers can implement a comprehensive approach integrating cultural practices, biological control, and targeted chemical interventions. This approach reduces reliance on broad-spectrum insecticides, minimizes environmental impact, and preserves beneficial insect populations [1]. This information provides an opportunity to maintain records of thrips populations and their dynamics over time. These records can serve as valuable historical data for tracking population trends, understanding pest pressure, and evaluating the effectiveness of control measures. Such documentation helps improve decision making in subsequent growing seasons. In summary, monitoring WFT populations in the field is crucial for early detection, informed decision-making, determining thresholds, implementing IPM strategies, optimizing control timing, managing resistance, and maintaining records. By actively monitoring thrips populations, growers can proactively manage these pests, protect their crops, and enhance their pest management practices’ overall effectiveness and sustainability.

Monitoring WFT populations requires reliable methods to assess thrips abundance and activity. There are two commonly employed methods for WFT monitoring.

1. Sticky traps are widely used to monitor thrips populations in the field [40]. These traps consist of yellow or blue sticky cards or tapes that attract and capture thrips; therefore, the trap color mimics the visual cues that thrips are naturally attracted to [41]. The traps can be hung at various heights within the crop canopy or placed near susceptible plant parts. Thrips are attracted to the traps, land on the sticky surface, and become trapped. The traps should be checked regularly (typically weekly) and replaced as needed [42]. These traps can also be amended with olfactory cues (e.g., allelochemical, pheromone, lure, or plant-based attractants specific to WFT) to be combined with visual cues to increase WFT attraction [43]. By counting the number of thrips captured on the traps, growers can estimate population levels, track population trends over time, and make informed decisions regarding pest management strategies.
2. Visual inspections involve direct observation of plants for signs of thrips activity, including feeding damage and thrips’ presence [19]. This measure requires careful examination of plant foliage, flowers, and fruits. Thus, growers should look for characteristic feeding damage such as stippling, silvering, scarring, or discoloration on leaves and other plant parts, and they should also observe the presence of adult thrips, larvae, or their eggs. Inspections should cover multiple locations within the field or greenhouse to ensure representative sampling. To enhance the accuracy of visual inspections, using a hand lens or magnifying glass can be helpful, as thrips are small and may not be easily visible to the naked eye [44]. Visual inspections should be conducted regularly, ideally at least once a week, especially during periods when thrips populations are expected to be more active or when crops are most vulnerable to thrips damage. It is worth noting that combining multiple monitoring methods, such as sticky traps and visual inspections, can provide a more comprehensive understanding of thrips populations and their behavior.

## 4. Effective Control Strategies to Manage Thrips Populations

Given the significant economic losses associated with WFT, growers and agricultural professionals strive to implement IPM strategies to manage thrips populations effectively. By combining cultural practices, biological control methods, targeted chemical interventions, and early detection measures, it is possible to mitigate the damage caused by WFT and protect crops from their devastating effects [2]. In this context, implementing effective control strategies to manage thrips populations is paramount for several reasons, such as crop protection, plant fitness, virus-based disease prevention, sustainable agriculture, and market access [45]. By employing IMP practices, early detection, and a combination of cultural, ethological, biological, and targeted chemical control methods, the WFT-derived damage can be effectively mitigated to ensure the long-term sustainability of agricultural systems. Therefore, understanding the current knowledge regarding control tactics is crucial for effectively implementing IPM schemes tailored to specific crop conditions. As pest management practices continue to evolve, it is essential to stay updated on the various control methods studied and developed.

### 4.1. Cultural Control Strategies

Crop rotation and diversification effectively disrupt the WFT life cycle and reduce infestation levels [46]. By implementing these practices, growers can create unfavorable conditions for thrips’ survival and reproduction. In addition, different crops have varying growth habits, architecture, and environmental requirements. Thus, crop diversification within a rotation alters the habitat and environmental conditions, making it less favorable for thrips [47]. For instance, planting taller or denser crops or plants less-susceptible to WFT can create a more challenging environment for thrips to locate suitable host plants and reproduce [48]. In fact, due to WFT relying on visual and olfactory cues to locate host plants, crop rotation and diversification can confuse them by altering the field’s spatial arrangement and odor profiles. This disruption hinders their ability to navigate and locate suitable host plants, reducing their overall infestation levels.

On the other hand, crop diversification can support the presence and activity of natural enemies, such as predatory insects or mites, which help control thrips populations. Different crops attract diverse natural enemies, and rotating crops can create a more diverse and stable ecosystem that favors the establishment and persistence of these beneficial organisms [47]. However, when implementing crop rotation for thrips management, it is essential to consider factors such as the duration of the crop rotation cycle, the susceptibility of alternative crops to thrips, and potential challenges related to market demands and crop profitability [49]. In this regard, it is highly important to adjust the planting timing to avoid peak periods of thrips activity. Depending on the specific crop and regional conditions, early or delayed planting can help minimize thrips populations by avoiding their peak reproductive and feeding periods. Additionally, plant density and proper canopy management techniques can improve air circulation and reduce humidity within the crop canopy. This can make the environment less favorable for thrips infestation and limit their population growth [6,50].

Trap cropping is another cultural strategy based on using plants highly attractive to WFT to divert their attention away from the main crop [51]. The trap crops act as sacrificial plants, attracting thrips away from the valuable crops and facilitating easier management [52]. Recently, a chemotactic-based ethological approach was employed to manage WFT populations [53]. Such an approach used an alarm pheromone (“Push”) to deter thrips from entering greenhouses and an aggregation pheromone (“Pull”) for mass trapping inside the greenhouses. To implement this method, the greenhouse fences were treated with a wax formulation of the alarm pheromone, while a yellow trap covered with sticky material containing the aggregation pheromone was deployed inside the greenhouses. The WFT aggregation pheromone was a mixture of two components, i.e., neryl methylbutanoate and lavandulyl acetate, whereas the WFT alarm pheromone (anal droplet) contained decyl acetate and dodecyl acetate. This strategy was effective in field trials as it significantly reduced thrips density in hot pepper flowers and the monitoring traps [53].

Furthermore, incorporating sanitation practices during rotation intervals helps reduce overwintering sites and alternative hosts, and minimizes the carryover of thrips populations between crops. Notably, the removal of crop residues, weeds, and alternate host plants can significantly reduce thrips populations, making it an essential aspect of effective thrips control [2]. Thrips tend to hide and reproduce in plant debris, so eliminating these potential breeding sites helps disrupt their life cycle. Moreover, proper irrigation practices, such as drip irrigation instead of overhead sprinklers, can help reduce thrips populations. And finally, using reflective mulches, such as aluminum or silver-colored mulches, can deter thrips by confusing their orientation and disrupting their ability to locate suitable host plants [54].

By integrating these cultural control strategies into an overall IPM program, growers can effectively manage WFT populations while minimizing reliance on chemical interventions. It is important to customize these strategies based on the specific crop, regional conditions, and the biology and behavior of WFT in the local area [15]. Regular monitoring and assessment of WFT populations are also critical to adjusting and fine-tuning cultural control strategies for optimal results.

### 4.2. Chemical Control Strategies

The selection of appropriate insecticides for WFT management is crucial to effectively control thrips populations while minimizing the impact on beneficial insects. Thus, some considerations should be taken into account when choosing insecticides. For instance, different insecticides vary in their efficacy against WFT. It is important to select insecticides that have demonstrated effectiveness specifically against thrips and to consider those having multiple modes of action to minimize the risk of resistance development in thrips populations, according to IRAC (insecticide resistance action committee) classification [41]. Using insecticides with different modes of action in rotation can help manage resistance and maintain long-term effectiveness.

There are several insecticides available for controlling WFT [1]. Choosing effective insecticides against thrips is important, as is considering their impact on beneficial insects and the environment. The most used insecticides are following listed [1]:

- Neonicotinoid insecticides, such as imidacloprid, acetamiprid, and thiamethoxam, are widely used for thrips control. They act on the nervous system of thrips, resulting in paralysis and death. However, neonicotinoids have been associated with adverse effects on pollinators and other beneficial insects, so their use should be carefully evaluated and limited to situations where alternative options are not feasible.
- Spinosad is derived from a soil bacterium and is effective against thrips. It acts on the nervous system, causing paralysis and death. Spinosad has low toxicity to many beneficial insects and is relatively considered a safer option.
- Pyrethroid insecticides, such as bifenthrin, cyfluthrin, and lambda-cyhalothrin, are commonly used for thrips control. They target the nervous system of thrips, resulting in paralysis and death. However, pyrethroids can adversely affect beneficial arthropods, including predatory mites and parasitic wasps.
- Organophosphate insecticides, e.g., malathion and chlorpyrifos, are effective against thrips. They disrupt their nervous system, leading to paralysis and death. However, organophosphates are broad-spectrum insecticides that can harm beneficial insects and pose risks to human health, so their use should be carefully considered.

Other chemical-based tactics have been recently evaluated, within the last three years, against WFT. Such evaluation comprises the mineral oil-based horticultural spray oil, PureSpray™ Green (PSG), and a sodium magnesium chlorophyllin photosensitizer formulation, SUN-D-06 PS [55], essential oils from bioactive plants such as *Zataria multiflora* Boiss [56], and ethanolic extracts from *Cercis siliquastrum* L., *Calendula officinalis* L., *Peganum harmala* L., and *Melia azedarach* L. [57]. In addition, other commercial insecticides (e.g., proteus (thiacloprid + deltamethrin) and pyridalyl [58], spirotetramat [59], chlorpyrifos, beta-cypermethrin, abamectin, and thiamethoxam [60]) and naturally occurring compounds (e.g., anisole [61], azadirachtin [62], matrine [58], and methyl benzoate [63]) have also been evaluated against WFT to see the direct and fumigant effects. These tested chemical treatments evidenced 100% mortalities at reasonable doses (i.e., <500 ppm) and LC_50_ values between 15 and 200 ppm.

### 4.3. Biological Control Strategies

A great deal of information has been published about the natural enemies of thrips, and this type of strategy has promoted several evaluations during the last five years. Bugs of the family Anthocoridae (Heteroptera) are among the most used natural enemies employed for the control of *F. occidentalis* with *Orius albidipennis*, *O. insidiosus*, *O. laevigatus*, *O. majusculus*, *O. minutus*, *O. niger*, and *O. tristicolor* being the most reported around the world [64], and even those occurred in Colombia, such as *O. championi*, *O. florentiae*, *O. fuscus*, *O. insidiosus*, *O. pele*, *O. thyestes*, *O. tristicolor*, *O. pumilio*, and *O. laevigatus* [65,66]. All these hemipteran bugs feed on thrips by piercing the host’s cuticle with its stylet and sucking the hemolymph. Avellaneda et al. monitored six crop farms in the Bogota Plateau, finding the presence of *O. insidiosus* and *O. pumilio* [66]. These two species, possibly sympatric [67], naturally occurred in areas with vegetation neighboring the greenhouses, which shows their ability to adapt to these highly intervened agroecosystems where the presence of pesticides is frequent. The capacity of settlement inside crops is a desirable characteristic for predatory species to achieve thrips control, but also in the areas between greenhouses where thrips move in search of new colonization areas. Mass rearing of natural enemies at affordable costs is essential to implement the use of natural enemies in commercial areas. In 2015, Avellaneda et al. performed life table studies founding that *Sitotroga cerealella* eggs are a suitable diet for mass rearing of *O. insidiosus* [68]. In contrast, a mixed diet composed of individuals of *F. occidentalis* and eggs of *Sitotroga cerealella* allows a better rearing performance of *O. insidiosus*, and a feeding frequency of 48 h allows the achievement of 4.2 times more individuals than a 24 h feeding frequency [69].

Recently, neuropterans predators of the family Chrysopidae as *Chrysoperla comanche* and *C. externa* have been reported as effective predators of *F. occidentalis* on tomatoes grown under glasshouse conditions, when a large number of eggs are released six days before the f blossom. However, it is necessary to determine the appropriate release ratio of predators with respect to WFT biological stages by some sampling units [70]. The competition effects that could arise between the two *Chrysoperla* species are also unknown. There are no previous reports of Chrysopidae as control agents for thrips, which opens the possibility for research about this topic even in Colombia, where several species of *Chrysoperla* are commercially available for control of other pests.

Several species of mites have been used as a biological control for thrips. *Hipoaspis miles* and *Gaeolaelaps* (*Hipoaspis*) *aculeifer* have been reported as natural enemies of the edaphic phases of *F. occidentalis* feeding on beans, with *G. acuelifer* being the most efficient under laboratory conditions [71]. This soil-dwelling predatory mite was reported in rose crops in the Bogota Plateau in 2016, and being Colombian population has biological attributes that make it a good candidate for biological control [72]. Additionally, the control effect of *Phytoseiulus persimilis* can be related to non-consumptive effects that increase mortality and decrease WFT oviposition [73]. Generalist predators like *Balaustium leanderi* are also promising as a control strategy since they feed on more than one pest species, which also allows them to establish in crops more readily [74,75].

A combined control strategy based on releases of *Amblyseius swirskii* and *O. laevigatus* was effective in sweet pepper commercial greenhouses in Hungary [76]. Moreover, Wiethoff evaluated the combination of the predatory mite *Amblyseius cucumeris* and soil-dwelling predatory mites for the WFT control [77], although in other cases, the simultaneous release of several predators has not shown a better result than those obtained with releases of each predator alone [78]. It highlights the importance of making this type of evaluation under specific agroecological conditions and using local natural enemies.

Entomopathogenic nematodes are a widely known control strategy for WFT, and several studies have demonstrated their anti-WFT capacity [79,80,81]. A combined biological control strategy based on using the predators *Orius* spp., *Ambliseius* spp., and *Hetororhabditis bacteriophora* has been proposed as promising [82]. Similarly, the combined use of *Heterorhabditis bacteriophora* and the predatory mite *Amblyseius cucumeris* effectively suppressed the WFT population density [83]. The entomopathogenic fungus *Beauveria bassiana* formulated in granules, applied to soil—the most common formulations are oil-based suspensions and water-based powders—was found to be effective in controlling the soil-dwelling stages of *F. occidentalis* in glass house tomato and cucumber crops [84] and greenhouse eggplant crops [85], and could also be evaluated in ornamental commercial crops in Colombia. Combinations of entomopathogenic fungus (Hypocreales: Cordycipitaceae) and the rove beetle (Coleoptera: Staphylinidae) for suppressing WFT populations under greenhouse conditions could provide a complementary control action on their different developmental stages, presenting a relevant topic for future research [86].

Double-stranded RNAs (dsRNAs) have recently emerged as promising tools for RNA interference (RNAi)-based pest control, including for managing WFT [87]. RNAi is a natural biological process that regulates gene expression by degrading specific messenger RNAs (mRNAs), thereby silencing the target gene. In the context of controlling WFT, researchers have identified and targeted essential genes in thrips’ biology using dsRNAs [88]. When introduced into thrips through feeding or other delivery methods, these dsRNAs trigger an RNAi response, leading to the degradation of the corresponding target gene’s mRNA. As a result, the expression of the essential gene is suppressed, disrupting vital physiological processes, and ultimately impacting the thrips’ survival, development, or reproduction.

A challenge in utilizing RNAi as a control strategy for thrips is ensuring the effective delivery of dsRNAs. Thrips’ small size and external feeding behavior make it necessary to explore innovative delivery systems, such as incorporating dsRNAs into plant tissues or developing dsRNA-coated substrates that can be attractive to thrips. Additionally, target specificity must be considered carefully, as unintended effects on non-target organisms must be minimized [88]. Despite these challenges, research on the use of dsRNAs to manage WFT shows promising results in laboratory and greenhouse settings [89]. The development of RNAi-based products for field applications could offer a more sustainable and environmentally friendly approach to thrips control, reducing the reliance on traditional chemical insecticides. Hence, as with any emerging technology, further research is needed to optimize dsRNA delivery and ensure its efficacy under field conditions. Nevertheless, the potential of RNAi-based strategies in controlling WFT and other agricultural pests represents an exciting frontier in pest management research [89].

As an overview of recent research efforts, Table 1 contains a comprehensive summary of various studies performed in the last five years, focusing on biological control strategies against WFT. The table encompasses investigations on the utilization of nematodes and entomopathogenic fungi, as well as the deployment of predators such as bugs and mites. Furthermore, it includes studies involving the application or transfer of RNA interference (RNAi) technology through microorganisms to genetically modified plants to combat WFT infestations. In Table 1, each study is listed, accompanied by key findings and outcomes. The compilation underscores the growing interest in exploring environmentally friendly and sustainable approaches to manage WFT populations while minimizing the dependence on conventional chemical pesticides. The array of biological control agents investigated reflects the diverse strategies employed to target different life stages and biological vulnerabilities of WFT. Through this comprehensive summary, researchers and practitioners in the field of pest management gain valuable insights into the potential efficacy and challenges of employing biological control methods against WFT. It serves as a valuable resource for understanding the current state of research in this area and identifying potential avenues for further investigations to refine and optimize these alternative pest management strategies.

### 4.4. Ethological Control Strategies

Ethological control, also known as behavior-based control, offers an innovative and promising approach to managing WFT populations. This strategy leverages the understanding of thrips’ behavior and ecological interactions to develop applicable control methods [2,12]. By targeting specific behaviors and exploiting their natural tendencies, ethological control aims to disrupt critical life processes and reduce the impact of WFT on crops. Two ethological control tactics comprise the exploitation of semiochemicals (i.e., chemical signals involved in communication between organisms) and visual stimuli. In the case of WFT, chemical and/or visual cues (e.g., pheromones and colored materials, respectively) have been explored to alter their behavior, luring thrips into traps and, ultimately, leading to population reduction.

#### 4.4.1. Volatile Chemical Attractants

The report by Teulon et al. [121] is one of the first works about volatile attractants showing effectiveness for capturing thrips under field and greenhouse conditions. The compounds ethyl nicotinate, *p*-anisaldehyde, and benzaldehyde allowed for the increased capture of thrips inhabiting flowers and belonging to several species up to 35 times. However, when *p*-anisaldehyde was added to sticky traps under greenhouse conditions, the capture of adult females of WFT increased to a lesser extent (1.8 to 6 times), which according to the authors, could be in part explained by increased captures in traps without attractant which possibly was placed inside the plume of near traps with attractant. An important approach from this work is that although displacement of thrips happens in part randomly, olfactory and visual stimuli play an important role, as can be deduced from the differential captures obtained when they are exposed to different olfactory and visual cues, and such behavioral responses could depend on genetics, physiological state, and experience. This research also reports that compounds *p*-anisaldehyde and benzaldehyde are present in flowers of numerous plant species, which are common hosts of thrips inhabiting flowers, while such compounds were not attractive for species of cereal thrips. Teerling et al. identified and demonstrated the bioactivity of a two-component alarm pheromone from *Frankliniella occidentalis* composed of decyl acetate and dodecyl acetate in proportion 1.5:1 [122], although the response off individuals was weak; therefore, the authors suggest that its use for control strategies would be limited.

Evaluations of the attractiveness of plant volatiles in olfactometer made by Koschier et al. have confirmed the attractiveness of compounds *p*-anisaldehyde and benzaldehyde [123] and identified attractant activity of other compounds such as *o*-anisaldehyde, the monoterpenes geraniol, nerol, linalool, and (+)-citronellol, the sesquiterpene (E)-β-farnesene, eugenol, and 3-phenylpropionaldehyde, two phenylpropanoids, and a nonfloral odor: ethyl nicotinate. The compound 1,8-ceneole also has been reported as an attractant of thrips by Katerinopoulos et al. [124]. Some pyridine compounds, mainly methyl and ethyl isonicotinates, have been reported as attractive under field conditions when used as lures in yellow sticky traps [125], increasing catches by around fourteen times with respect to the control. The studies by Teulon et al. [126] and Davidson et al. [125] were the basis for the development of Lurem TR^®^, a commercial lure produced by Koppert and reported as effective for the monitor and control of several thrips’ species, among them *F. occidentalis*. Koschier et al. preliminarily reported the compound salicylaldehyde as a possible repellent [123], and subsequently, the repellent effects of salicylaldehyde and the compound methyl salicylate were confirmed [127], previously reported by Chermenscaya et al. [128], who reported that females avoid cucumber leaf discs treated with this compound. While salicylaldehyde induced avoidance for up to three hours, the effect of methyl salicylate lasted for five hours. Regarding deterrence, salicylaldehyde did not show an effect, while methyl salicylate prevented oviposition and reduced damage. The authors consider that aerial application of repellent compounds could be used as a defense against herbivory, although in *F. occidentalis*, part of the behavioral responses induced by repellents implies increased flight activity, which could trigger dispersion of TSWV in crops. Furthermore, a mixture of *p*-anisaldehyde and ethyl nicotinate elicits positive antennal and behavioral responses in WFT at concentrations (0.1, 1, 10 g/L) through depolarization, attraction, and successful trapping under greenhouse conditions [129].

This problem was also foreseen by McDonald et al. [130] regarding the possible use of the alarm pheromone as a “push” component in the design of a “push-pull” strategy for the control of thrips. Additional research about the feeding behavior of *F. occidentalis* after alighting is needed to better understand the possible results of such applications. An aggregation pheromone produced by males and attracting individuals of both sexes has also been reported [20,131,132]. This aggregation pheromone is composed of (*R*)-lavandulyl acetate and neryl (*S*)-2-methylbutanoate, and showed the capability of increasing the catches of individuals between 38–84% on yellow sticky traps when the compounds were used in a 1:1 blend or employed neryl (*S*)-2-methylbutanoate alone [20], having the advantage of doses of chemical much lower than the reported previously by Teulon et al. [121]. This aggregation pheromone is the basis for developing the commercial lure Thripline-AMS^®^, produced by Syngenta Bioline^®^.

Pollen is a main component in the diet of thrips, which has led to investigating the possible attracting effect of volatile compounds present in pollen, particularly from pine trees, since it has been established that they meets nutritional conditions that favor the biological parameters of this insect. Through this approach, Abdullah et al. [133,134] identified the attractive properties of the compound (*S*)-(–)-verbenone in olfactometer essays and field conditions using sticky traps. These authors report that sticky traps baited with 1% verbone allowed captures of thrips similar to those observed in traps baited with the commercial lures Lurem TR^®^ and Thripline-AMS^®^. Binyameen et al. [135] reported a synergistic effect of eugenol when blended with ethyl isonicotinate, which increases attraction by 100% compared to that obtained using ethyl isonicotinate alone in the species *Thrips tabaci*. Additional research is needed to determine if a similar effect is also maintained in the species *F. occidentalis*. Additionally, pathogen-induced plant volatiles (PIPVs) have also been studied; WFT was attracted to both TSWV- and tomato yellow leaf curl virus (TYLCV)-infected plants and showed no preference between plants infected by either virus, which released mostly humulene, caryophyllene, δ-elemene, and β-copaene [136].

In Colombia, a recent study was performed by Avellaneda et al. on WFT attractants produced by rose plants [137]. The approach of such exploration was identifying attractants in the volatile organic compounds (VOCs) profile produced by commercial host plants widely attacked by the pest under local conditions, particularly rose varieties. The authors initially selected a sample of six cultivars, presenting three of them with a high preference for thrips and the others having a low preference for the insect in Y-olfactometry essays [137]. Headspace-solid phase microextraction (HS-SPME) was used to collect the volatile compounds directly from rose plants in the greenhouse commercial crops. Nine compounds presenting an association with a high incidence of thrips were identified employing the Cramer’s phi coefficient, and then its attractant capacity was evaluated by linear olfactometry. Six of such compounds elicited a response of attraction in the thrips, i.e., (±)-theaspirane, nonanal, (*Z*)-3-hexenyl acetate, β-caryophyllene, 2-phenylethyl acetate, and caryophyllene oxide. New attractants could be developed from these compounds to be used under field conditions, and the already developed attractants could be improved.

#### 4.4.2. Visual Stimuli

Control strategies that exploit the color perception and attraction behavior of WFT have been developed and implemented in diverse crop systems. These approaches capitalize on WFT’s ability to perceive certain colors and their tendency to be drawn toward them. The preference of *Frankliniella occidentalis* for white traps was initially reported [138], obtaining 91% of total caches with that color and only 8.9% on yellow traps in pear orchards. A similar result was subsequently reported [139], finding 57% of captures of *Scirtothrips citri* occurred in gloss white traps, 31% in yellow primrose traps, and 12% with other colors in a Navel Orange grove. The WFT preference for white color was confirmed by Yudin et al. [140] in lettuce farms when compared with other 14 colors. Chu et al. evaluated the effect of nine colors on catches in CC traps placed in diverse crops [141], discovering that true blue and white were the most attractive for *F. occidentalis*. The results by Demirel and Cranshaw contrast with the previous reports [142] since neon yellow, orange, neon orange, and neon pink were found as the most attractive colors in cabbage crops, while white and blue were not attractive; it was attributed to the fact that previous evaluations were made in different crops. A preference for yellow flowers has been reported in experiments conducted in chambers containing flowers of different colors [143]. In this study, the visible and near-infrared spectra of flowers and sticky cards were compared in search of common characteristics between both attraction sources, finding that yellow *Gerbera jamesonii* flowers and yellow sticky cards share a similar reflectance spectrum ranging from 500–600 nm, therefore, the authors recommend the use of yellow sticky cards over blue and white ones. Larrain et al. evaluated different sticky traps colors in *Capsicum annum* crops obtaining high preference by blue, white with a blue strip, and white traps over yellow if weekly catches were over 300 individuals [144]. A high preference for blue sticky traps respect to white and yellow ones was also found by Joyo and Narrea in grape vine crops [145].

Additionally, blue traps were found to be more attractive than yellow in herb and *Alstroemeria* greenhouses [146]. Changes in resource availability could explain the discrepancies observed in the preferred color in different reports as plants go through different stages of development, resulting in different responses of thrips to spectral cues [147]. According to Gillespie and Vernon [148], besides color itself, reflectance at different wavelengths could be important: landing of thrips in yellow traps could be promoted by a wavelength-specific reflectance higher than 80% for wavelengths between 550–650 nm and low UV reflectance, around 20%. Johansen et al. [146] explain the lower catches obtained in yellow traps to a low reflectance (50–55%) in the 570–650 nm range. Shape, size, background, positioning angle, and tridimensional design also affect the traps’ performance for insects capture [149]. Different shapes of flat traps and background colors have also been evaluated under laboratory conditions [150], e.g., circular traps allowed captures 2.7 and 1.5 times higher than square traps, and the black background also increased captures with respect to a yellow, blue, or green background. When this design was evaluated under field conditions, the catches of *F. occidentalis* were 2.3 to 2.1 times higher than those obtained with commercial square yellow sticky traps. Broughton et al. [38] evaluated the effect of six colors on the capture of thrips and beneficial insects, finding twice as high catches of *F. occidentalis* on blue and seven times higher catches on the commercial Seabright blue^®^ traps concerning yellow traps.

Evaluations made in blackberry crops evidenced higher captures of *F. occidentalis* in blue traps compared to yellow, white, black, green, and violet ones when the crop was placed in tunnels, but the results changed for open field crops, obtaining higher catches in yellow traps, which could be explained by a lower sunlight penetration in tunnels [151]. In addition, the preference of thrips by trap colors could be influenced by attributes of the plant [146], whose fact could explain a higher preference of *F. occidentalis* by blue traps over yellow when tested in white flower *Alstroemeria* varieties for tests realized in colored varieties. Such differences could be rationalized by integrating visual and olfactory cues mediating the attraction of thrips. The effect of white, red, green, purple, yellow, and blue trap colors, flower-like, circular, rectangle, and triangle trap shapes, and different olfactory cues was evaluated by Ren et al. [43]; they found a higher WFT preference by yellow under laboratory conditions, and by blue under field conditions, attributed to the presence of a black background in the laboratory, which could provide a high contrast enhancing the attraction towards yellow traps and a lower light intensity also in the laboratory. Yellow was selected for shape and attractants evaluations, finding that a flower-like shape is the most attractive for thrips. Although the selective response to visual cues reduces from 30-cm onwards, such response is enhanced using olfactory cues, particularly the volatile extract of flowering *Medicago sativa* and *p*-anisaldehyde.

The cited studies have been based on the response induced by the light reflected from traps. Some recent works have centered on other physical mechanisms such as fluorescence and light emission. Fluorescent yellow traps were found more attractive than non-fluorescent yellow and blue traps for *Thrips tabaci* and *F. occidentalis* in cabbage crops [152], which was attributed to the low reflectance of fluorescent yellow traps in the UV, since it has been proved repellent for thrips [153]. In this context, Johansen et al. [146] evaluated the phototactic response of *F. occidentalis* to yellow and blue sticky traps with blue light emitting diodes LEDs in colored and white *Alstroemeria* varieties, obtaining for blue traps catches 3.4 and 4.0 times higher than those observed in blue traps without LEDS in one of the experiments realized. Although in yellow traps with LEDS, catches increased 4.5 times concerning yellow traps without LEDs, they were only slightly higher than the obtained in blue traps without light and lower than the observed for blue traps with LEDs, which is possibly due to a higher stimulus produced by the reflection of the blue light in the blue sticky traps, while in the yellow traps the blue light is mostly absorbed. Liu et al. [154] evaluated under laboratory conditions the effect of LEDs of eight different light spectra associated to specific wavelength values (red, orange, yellow, green, blue, violet UV, white), two levels of incident illumination (6000 and 12,000 lx), and two levels of radiating energy (60 and 120 mW/cm^2^) in a multiple channel device on the visual selection of thrips, and found high selective responses to yellow, green, violet, and UV, and low selective responses to red, orange, and white light. Regarding incident illumination and radiating energy, only violet presented a significant change (reduction) in selective response when illumination is increased from 6000 to 12,000 lx. The same authors evaluated the approach sensitivity (i.e., the distance that individuals move to the proximity of the light source) of thrips to the same colors, founding that only yellow, green, violet and UV induced a response of approach sensitivity, although it was always higher for violet and UV with a light energy of 60 mW/cm^2^. The same authors point out the importance of additional research that allow the understanding of the responses of thrips to light spectra at a physiological level. Evaluations of the thrips response to specific light spectra of LEDs were also made by Stukenberg et al. [155], who found that *F. occidentalis* could have two specific behaviors controlled by two photoreceptors, induced by wavelength light corresponding to blue and green colors, and possibly a third UV photoreceptor could be important, configuring a three-chromatic photoreceptor system. However, such configuration could change throughout the developmental stage and sex of the insects. In the same study, using blue LEDs is recommended to increase catches of *F. occidentalis* in sticky traps, although green LEDs could be an alternative to increasing catches of a broader range of insect pests. Recent research suggested that some physical properties of glue used in traps could affect the attraction of thrips. Experiments conducted in wind tunnel and field by van Tol et al. [156] evidenced that using clear glue as D41 induced a marked preference of *F. occidentalis* by yellow traps over blue, but the preference was inverted when a diffuse (whitish) glue as Stikem was employed. A reduction in reflection of blue, violet, yellow, and green light when D41 is applied on yellow cards with respect to the reflection observed for Stikem was identified, but it is not clear to what point the differential response observed is due to the change in light reflection, and so is important to develop more research about the reflective properties of glues and its effect on catches.

Regarding the research of visual cues applied to the management of thrips in Colombia, the works have focused on the development of applied protocols for the monitorin and control with colored sticky traps, partly based on temperate zone reports [157,158,159]. One of the first reports was made by Cárdenas and Corredor [157], who evaluated rectangular traps of the colors white, purple, yellow, red, orange, and green in a commercial greenhouse of chrysanthemum and found higher catches of *F. occidentalis* in white, purple, and yellow traps. Medina et al. [158] evaluated the captures obtained using acrylic sticky traps of colors white, yellow, and three shades of blue in a commercial greenhouse of chrysanthemum, reporting that the blue color with a wavelength of 440 nm and reflectance of 53.58% allowed the highest captures in the traps. Recently, the microstructures present on the surface of rose petals (papillae) have been suggested to affect the WFT color perception since the size of papillae differs between attractive and non-attractive rose varieties [160], which could be helpful for the development of traps-based not only on true color but also on the modifications that microstructures could generate in the color perception of thrips.

### 4.5. Host Plant Resistance-Based Controls Strategies

This tactic has been little developed so far in pest management strategies, possibly due to the rise in chemical control. However, several recent investigations at a global level report advances on this topic, particularly in two types of plant responses, such as constitutive and induced defenses against WFT.

#### 4.5.1. Constitutive Defense against WFT

Several authors have reported mechanisms known as constitutive defense. In *Gossypium* sp., the differences in the abundance of thrips observed between genotypes have been attributed to traits of the morphological or biochemical kind, which can be present in the plant before the attack (constitutive) or induced by the presence of the pest. In some cases, abundant hairs on leaves have been assumed to be a resistant trait against thrips [161,162,163,164]. However, in other cases, glabrous leaves have been associated with resistance [165,166], which could be explained by the confounding effects of several traits changing between genotypes beyond the presence of hairs, and in most cases, reported resistance has not been clearly linked to specific morphological or biochemical traits [167]. A more consistent relationship between a morphological trait (papillae density) and resistance is reported by Wahyuni et al. [168] in *Gladiolus hybridus*, who compared the ^1^H NMR (proton nuclear magnetic resonance) profiles of thrips resistant and susceptible varieties expressing a wide range of variation in papillae density, and found that two unidentified triterpenoids saponins and the amino acids alanine and threonine presented relative leaf concentrations that were highly correlated between them and with papillae density, from which the authors suggest that papillae are involved in the production and storing of this compounds responsible for plant resistance. 

From research conducted on plants of the genus *Capsicum*, it has been possible to determine that the stage of development of the plant is an important aspect in the expression of the level of resistance present in the plant [169,170], and therefore, the screening of resistant genotypes for plant breeding purposes must consider the different stages of development of the plant, while the resistance level is not affected by the leaf position in the plant. On the other hand, it was not possible to establish a correlation between the resistance level to *F. occidentalis* and *Thrips tabaci* in this particular research. As in the case of *Gossypium* sp., the traits responsible for the resistance reported in *Capsicum* are yet to be solved. Since ornamental crops are among the most affected by the attack of *F. occidentalis* in Colombia, the possible resistance mechanisms in these crops are a topic of great interest. Gaum et al. [171] found rose cultivars with variable degrees of resistance, in some cases relatively high, to the point of being associated with negative values of the intrinsic rate of population growth ($r_{m}$), indicating the infeasibility of the pest population being able to establish itself in these genotypes, possibly due to antibiosis, although the specific mechanisms involved were not established. According to these authors, the odor of rose flowers did not attract *F. ocidentalis* but repelled them. However, recent research has demonstrated significant positive chemotaxis to compounds obtained from six rose cultivars [137]. On the other hand, the same authors reported antixenosis associated with cultivars with red and pink colored flowers, while the yellow ones were highly attractive. A similar result is reported in other studies [172,173], whose higher preference by cultivars with yellow and white flowers over those with red flowers. Flower attributes, different from flower color, could also be responsible for plant resistance and the mechanisms determining antibiosis and antixenosis are probably present in petal tissue but not foliar tissue [172]. Rigid petals that open widely could be associated with fewer food resources and less shelter [171]. Carrizo and Klasman [174] reported the differential preference of *F. occidentalis* by carnation varieties, being that those with red and dark flowers were less preferred, but the underlying causes are not evaluated. Host plant resistance has also been evaluated in *Chrysanthemum* during the vegetative period [175], founding a highly resistant variety, possibly due to antibiosis, although there is a need for additional research to establish the possible mechanisms of antixenosis. In the same crop, Kogel et al. [176] found a partially resistant cultivar in which the damage, insect reproduction, and adult survival were reduced for several populations of *F. occidentalis* in a lasting way through the evaluation time. However, possible mechanisms of resistance are still to be understood.

Developing varieties with plant resistance based on the color and morphology of the flower is difficult since the tastes and demands of markets must orient these traits. A significant advance in the achievement of resistance to *F. occidentalis* in crop plants is the development of transgenic potato plants expressing custom-made, multidomain proteinase inhibitors [177]. Herbivores produce enzymes with proteolytic activity (proteases) to digest the substrate they feed on. Plants, in turn, synthesize proteinase inhibitors (PIs) as natural defenses that inhibit the activity of such proteases [178]. According to the amino acid involved in the hydrolysis of peptide bonds, PIs are grouped into three classes: cysteine, serine, and aspartic proteases. Digestive extracts from WFT present an optimum proteolytic activity at pH 3.5 and can be inhibited by cysteine PIs. This can be evidenced by a substantial reduction in the oviposition of females fed on pollen diets from mixed origin treated with PIs [179]. From these results, Outchkourov et al. [180] developed transgenic potato plants over-expressing cysteine protease inhibitors and found that they are strongly deterrent to WFT, affecting the behavior of individuals as soon as one hour after feeding. Another significant advance in the knowledge of resistance mechanisms was the determination of chlorogenic acid and feruloyl quinic acid as a factor responsible for the resistance in some chrysanthemum varieties through in vivo and in vitro essays combined with NMR-based metabolomics. Chlorogenic acid reduces the amino acids’ bioavailability, which leads to a low digestibility of proteins. Chlorogenic acid also has activity against bacteria and viruses, allowing the possibility of breeding programs oriented to resistance to multiple pests and diseases [181]. The use of NMR-based metabolomics has allowed the identification of the presence of acylsugars as a resistance factor to thrips in wild tomato genotypes [182] and kaempferol glucoside in resistant *Senecio* hybrids [183].

#### 4.5.2. Induced Defense against WFT

Since induced plant defense mechanisms are regulated mainly by the phytohormones salicylic acid, ethylene, and jasmonic acid, elicitors with the capacity for activating the production of these compounds could be a way to activate such defense mechanisms [184]. This approach was employed by Chen et al. [185], who evidenced that infiltration of tomato plants with coronatine, a phytotoxin produced by the bacteria *Pseudomonas syringae*, enhanced the resistance of the plants against WFT through the increased production of polyphenol oxidases and proteinase inhibitors mediated by activation of the jasmonic acid signaling. Beyond the development of resistant genotypes, induced resistance could also be obtained by means of treatment of susceptible plants with appropriate elicitors, which could be not only of a chemical but also physical nature, such as UV radiation, whose effect modulating the constitutive and induced defenses to thrips in chrysanthemum plants has been reported [186]. Regarding chemical elicitors, life table studies of *F. occidentalis* on Kidney bean plants sprayed with a solution of CaCl_2_ presented a lower intrinsic rate of increase (r) and reduced preference concerning control plants, although additional research under field conditions is required [23]. In a similar way, Murata et al. [187] reported that the pretreatment of tomato plants with α-ionone, reduced the survival rate of WFT, promoting the expression of defense-related genes, different from those involved in the production of jasmonic acid or loliolide, two compounds associated with resistance to herbivory [188]. Judging from the available literature, research about plant resistance to thrips in Colombia has been very scarce. Resistance of cassava to *Franklinella* sp. and *Corynothrips stenopterus* was evaluated in 1254 clones from the International Tropical Agriculture Center (CIAT) by Schoonhoven et al. [189], who found 20% of accessions with no symptoms of the damage and attributed the resistance to the pubescence in leaves. This trait tends to disappear when the plant flowers, which reduces, in turn, the resistance of plants in this phenological stage. Frei et al. [190] performed QTL mapping of resistance to *Thrips palmi* in Common bean in Valle del Cauca, Colombia.

## 5. Outlook

The perspectives for managing WFT and developing effective control strategies hold both challenges and opportunities [2,191]. As agricultural practices and pest management approaches continue to evolve, several key areas offer potential advancements in WFT control. Therefore, integrating multiple control strategies, including cultural, biological, and chemical methods, will likely play a pivotal role in WFT management. Thus, continued research and refinement of IPM practices will lead to more efficient and sustainable control measures tailored to specific crops and regional conditions. Accordingly, breeding and developing WFT-resistant crop varieties holds significant promise for reducing thrips damage and exploring constitutive and induced plant defensive response against WFT. By identifying and incorporating genetic traits that deter thrips feeding and reproduction, researchers can enhance crop resilience and minimize the need for chemical interventions. These advancements should be aligned with precision agriculture, remote sensing, and sensor technologies, which offer opportunities for the real-time monitoring and early detection of WFT infestations. Timely and accurate information about thrips populations can enable proactive decision-making and targeted interventions, reducing the risk of economic losses. In addition, climate change may influence the distribution and behavior of WFT populations. Understanding these potential shifts and their impact on WFT dynamics is crucial for developing adaptive control strategies. Researchers are studying the effects of changing environmental conditions on WFT biology, phenology, and range expansion to inform future management approaches.

Similarly, the deepening and application of RNAi-based strategies for WFT control constitute great potential. Ongoing research aims to optimize the delivery systems and target specificity of dsRNAs, enabling more precise and effective suppression of essential WFT genes. This technology could provide a sustainable and environmentally friendly alternative to traditional chemical insecticides. Furthermore, continued collaboration among researchers, growers, industry stakeholders, and policy makers is vital for advancing WFT control strategies. Sharing knowledge, best practices, and successful case studies will facilitate the adoption of effective management techniques and encourage innovation in pest management.

It is important to note that the outlook for WFT control strategies will require a holistic and multidisciplinary approach. Integrating various tools and techniques, adapting to changing pest dynamics, and promoting sustainable practices will be critical for managing WFT effectively while minimizing environmental impact. By embracing innovation, collaboration, and the principles of integrated pest management, we can work towards a more resilient and sustainable future in WFT management.

## 6. Concluding Remarks

Recent advances in control strategies against WFT have shown great promise in improving the management of this agricultural pest. Several notable developments have emerged, providing new tools and approaches for effective WFT control. In this regard, integrating biological control methods, such as using natural enemies like predatory bugs, mites, and entomopathogenic fungi, has demonstrated encouraging results. Similarly, ethological control by chemical and visual stimuli has attractive potential to be developed, comprising more detailed information about WTF ethology. Combined or alone, these strategies offer environmentally friendly alternatives to chemical insecticides, reducing reliance on synthetic inputs and promoting sustainable pest management practices. Furthermore, the application of RNA interference (RNAi) technology has opened new avenues for the targeted control of WFT since, by utilizing dsRNAs that can silence essential genes in WFT, RNAi-based approaches can potentially disrupt key physiological processes and reduce thrips populations. Ongoing research aims to refine delivery systems and enhance target specificity to optimize the efficacy of this innovative control strategy.

On the other hand, host plant resistance has emerged as a valuable tool in WFT control. Breeding and developing crop varieties with inherent resistance to thrips feeding and reproduction traits offers long-term solutions for reducing WFT damage. Moreover, resistance induction through elicitors is another pertinent control alternative. By leveraging genetic resistance, growers can enhance crop resilience and reduce the need for chemical interventions. However, challenges remain, such as the development of resistance in WFT populations to control agents and the need for sustainable and economically viable approaches. Continued research, innovation, and adaptation to changing environmental conditions will be crucial for staying ahead of evolving WFT populations and ensuring long-term success in managing this pest. Understanding the various tactics can improve the scene to mitigate WFT damage, reduce reliance on chemical pesticides, and foster more sustainable agricultural systems.

## Figures and Tables

**Table 1:** Recent studies on biological control for WFT.

Scale ^a^	Product/Agent	Effect on Thrips	Ref.
L/G	dsRNA of TLR6 and CopE (transgenic *S. lycopersicum* to deliver RNAi)	>50% mortality	[90]
G	*Phytoseiulus persimilis* and *Amblyseius swirskii* (20 and 100 mites per m^2^)	>60% suppression	[91]
G	*Beauveria bassiana* dispersion by *Bombus impatients*	>75% suppression	[92]
L/M	*Neoseiulus cucumeris* (100 adults per m^2^)	84.5% suppression (larvae)	[93]
G	*B. bassiana* applied to *Tagetes patula* (L.), combined with *Neoseiulus* (=*Amblyseius*) *cucumeris* in slow-release sachets	Higher WFT number in marigolds than on crop plants.	[94]
G	Spraying dsRNA specific to vATPase-A or vATPase-B	100% mortality	[95]
L	Leaf disc-mediated dsRNA delivery system (antagomir and agomir)	>45% mortality	[96]
L	Pre-inoculation of *Orius sauteri* on host plants	Reduction in survival and reproduction	[97]
L	Symbiont mediated RNAi (SMR)—(BFo1 and BFo2)	Competition for bacterial colonization	[98]
L	Plastid-mediated RNA interference (PM-RNAi)	Efficient suppression/high insect mortality	[89]
G	Predatory mite *Anystis baccarum*	Reduction in feeding damage and population density	[99]
L	*Orius strigicollis*	T influences demographic traits and predation rates	[100]
L	Feeding solution of dsRNA specific to vATPase-B	>70% mortality	[101]
L	*A. swirskii*, *Transeius montdorensis*, *Amblydromalus limonicus*	>70% reduction population	[102]
L	*N. cucumeris* and *A. swirskii*	>60 reduction population	[103]
L	BotaniGard^®^ (a commercial form of *B. bassiana* strain GHA)	>80 reduction population	[104]
L/F	*Metarhizium anisopliae* CQMa421	LT_50_ = 5.5 days; 50–70% reduction	[105]
L/G	*Orius insidiosus* predation	>90% reduction population (4 bugs per plant)	[106]
L	*A. swirskii* as predator	Suppression and reduced plant damage	[107]
G	Granular formulation of *B. bassiana* + *Stratiolaelaps scimitus*	Suppression and reduced plant damage	[108]
L	*O. insidiosus* as predator	Thrips deposit non-volatile semiochemicals used by predator during foraging	[109]
L	*Dalotia coriaria* (predator–prey 1:15)	Reduced thrips density in sticky traps	[110]
L	*O. insidiosus* and *O. tristicolor*	Females preferred the flowering strawberry plants over the flowering sweet pepper plants	[111]
L	Predation of *D. coriaria* (three adults per 15.2-cm container)	Reduced thrips density in sticky traps	[112]
G	Entomopathogenic nematode *Steinernema yirgalemense*	53% mortality	[113]
G	Predation of *A. swirskii* and *T. montdorensis*	Release alternation (*T. montdorensis* in winter and *A. swirskii* in spring) to reduce population density	[114]
G	*Orius albidipennis*, *Macrolophus caliginosus*, *Chrysoperla carnea*, and *Trichogramma euproctidis*	Reduced population density	[115]
G	*A. limonicus* as predator	Low rearing T improve biological control function	[116]
G	MON 88,702 cotton expressing Bt toxin	Reduction of oviposition and larval developing	[117]
G	Synergistic Interaction of *Metarhizium flavoviride*, imidacloprid, and Diatomaceous Earth	>75% mortality	[118]
L	Feeding dsRNA of vATPase-B sequence	>80% mortality	[88]
L	*B. bassiana* ERL836 and JEF-007	ERL836 infected thrips easily	[119]
L	Entomopathogenic nematodes (*Steinernema yirgalemense*, *Heterorhabditis baujardi*, and *Heterorhabditis bacteriophora*)	>60% infection	[120]
L	*Gaeolaelaps aculeifer* as a predator	Predation rate = 2.6	[72]

^a^ Trial scale = L: Laboratory; G: Greenhouse; M: microcosm; F: Field.

## Data Availability

The data presented in this study are available on reasonable request from the corresponding author.
